# The Airway Microbiome-IL-17 Axis: a Critical Regulator of Chronic Inflammatory Disease

**DOI:** 10.1007/s12016-022-08928-y

**Published:** 2022-03-11

**Authors:** Jenny M. Mannion, Rachel M. McLoughlin, Stephen J. Lalor

**Affiliations:** 1 School of Biochemistry and Immunology, Trinity Biomedical Sciences Institute, Trinity College Dublin, Dublin, Ireland; 2 UCD School of Medicine, Conway Institute of Biomolecular and Biomedical Research, University College Dublin, Dublin, Ireland

**Keywords:** IL-17, Th17 cells, Respiratory tract, Airway microbiota, Chronic inflammatory disease

## Abstract

The respiratory tract is home to a diverse microbial community whose influence on local and systemic immune responses is only beginning to be appreciated. Increasing reports have linked changes in this microbiome to a range of pulmonary and extrapulmonary disorders, including asthma, chronic obstructive pulmonary disease and rheumatoid arthritis. Central to many of these findings is the role of IL-17-type immunity as an important driver of inflammation. Despite the crucial role played by IL-17-mediated immune responses in protection against infection, overt Th17 cell responses have been implicated in the pathogenesis of several chronic inflammatory diseases. However, our knowledge of the influence of bacteria that commonly colonise the respiratory tract on IL-17-driven inflammatory responses remains sparse. In this article, we review the current knowledge on the role of specific members of the airway microbiota in the modulation of IL-17-type immunity and discuss how this line of research may support the testing of susceptible individuals and targeting of inflammation at its earliest stages in the hope of preventing the development of chronic disease.

## Introduction

The human body is covered at all exposed and barrier surfaces with a panoply of bacteria, archaea, fungi and viruses. However, the true extent of their influence on host health and pathology has only recently been appreciated. Bacteria are the most numerous microbial components of the commensal microbiota and research to date has primarily focused on the bacteria that colonise the gastrointestinal tract. Significant advances have been made in our understanding of the crucial role these microbes perform, from regulating the development of the host immune system to the normal wiring of the brain [1–4]. Other mucosal sites too are colonised by commensal microorganisms that play critical roles in homeostatic functioning and defence against pathobionts. Once thought a sterile microenvironment, recent advances using culture-independent technologies have identified several bacterial species in healthy human lungs and growing evidence indicates that the upper and lower airways may be an important site for microbial regulation of immune responses, both locally and systemically. In the past decade, connections have been made between the composition of the respiratory tract microbiota and the pathophysiology of chronic inflammatory diseases such as asthma [5], chronic obstructive pulmonary disease (COPD) [6], cystic fibrosis (CF) [7] and rheumatoid arthritis (RA) [8]. In fact, changes in the relative abundance of only a small group of respiratory tract bacteria have repeatedly been associated with a range of chronic inflammatory conditions and a clear finding in many of these investigations is the centrality of IL-17 signalling in response to perturbations of this microbial community. IL-17 expression and neutrophil recruitment are typically associated with early inflammatory events [9], and it might be that altered abundance of these microbes promotes acute IL-17-type inflammation in the airways which, left unchecked, can become chronic and lead to major tissue destruction and fibrosis. Furthermore, IL-23-stimulated Th17 cells exert pleiotropic effects that extend beyond IL-17 expression and may also contribute to inflammatory events in these complex diseases. Here, we highlight the emerging association between IL-17-type immunity and bacteria that commonly colonise the human airways and discuss how this may contribute to the aetiology of chronic inflammatory disease.

## The Airway Microbiota

After the gastrointestinal tract, the respiratory tract bears the largest exposure to the external environment. At 70 m^2^, the surface area of the lungs alone is forty times that of the skin (1.8 m^2^), owing to the physiological arrangement of the bronchi and alveoli [10]. We breath in approximately 11,000 L of air daily, containing both particulates and microorganisms [11]. As such, the respiratory tract is a major site of microbial exposure and colonisation. Indeed, the respiratory tract microbiota comprises over 600 individual bacterial species that form distinct communities from site-to-site, each adapting to the diverse surfaces and physiological conditions (e.g. temperature and pH) that exist along its length [12, 13].

The anterior nares are lined with keratinizing squamous epithelial cells and, due to the presence of sebum, host a lipophilic bacterial community that closely resembles that found on the skin, including *Staphylococcus*, *Propionibacterium* and *Corynebacterium* species [14]. Lined with columnar ciliated epithelial cells and non-ciliated mucus-secreting goblet cells, the nasopharynx comprises a diverse aero-anaerobic bacterial community comprising *Streptococcus*, *Rothia, Veillonella, Prevotella* and *Neisseria* [15]. The oral cavity is home to a varied microbial community in which *Streptococcus, Haemophilus, Neisseria, Actinomyces, Prevotella* and *Veillonella* species are commonly identified in healthy individuals [16]. The oropharynx is lined with a non-keratinizing squamous epithelium and its bacterial community is characterised by the presence of *Neisseria*, *Rothia, Prevotella*, *Veillonella, Fusobacteria* and *Leptotrichia* species [13, 17–19]. Finally, the healthy lungs—once thought to be sterile in the absence of infection—harbor a diverse microbial community, partly resembling that which is found in the mouth [12]. The bronchial tree is lined with ciliated columnar epithelium that soon transitions to the low cuboidal epithelium of the respiratory bronchioles and, ultimately, alveolar epithelium that is specifically adapted for gas exchange [13]. A thin layer of surfactant contributes to alveolar integrity by reducing surface tension at the air–liquid interface. In addition, surfactant displays bacteriostatic effects and surfactant proteins bind to non-host oligosaccharides and promote leukocyte recruitment and phagocytosis [20]. Advanced sequencing technologies have identified *Prevotella* and *Veillonella* as the dominant genera in the trachea, bronchi and bronchioles [5]. *Streptococcus*, *Haemophilus*, *Moraxella*, *Pseudomonas, Fusobacterium* and *Porphyromonas* taxa are also commonly identified in this dynamic community that results from microaspiration of bacteria and other microbes in oropharyngeal secretions, and ongoing outward movement through mucociliary clearance and the cough reflex [5, 6, 21]. Immune surveillance by alveolar macrophages (AM) also contributes to the turn-over of pulmonary symbionts.

The importance of the microbiota in lung development was revealed by the significantly reduced number and structure of alveoli, and decreased mucus production, in GF mice compared to conventionally-housed mice or their wild counterparts [3]. In addition, the bacterial community in the airways regulates mucosal immune responses against respiratory infection and may have a role in the development of certain forms of lung cancer [22–25]. We are only beginning to understand, however, the impact of this airway microbiota on resident and circulating host cells, particularly T lymphocytes that orchestrate the pathophysiology of many chronic inflammatory diseases. For a long time, inflammatory diseases were thought to associate with either an IFNγ-driven Th1-type inflammatory response (e.g. multiple sclerosis) or a Th2-type response characterised by the expression of IL-4, IL-5 and IL-13 (e.g. asthma). However, the discovery in 2005 of Th17 cells, a distinct lineage of CD4 T helper cell that preferentially produced the inflammatory cytokine IL-17A, was a significant advance in our understanding of the pathophysiology of many autoimmune inflammatory diseases [26]. Since then, a central pathogenic role for IL-17-type immunity in the development or exacerbation of many chronic respiratory diseases has also become apparent, with a growing body of evidence linking activation of these cells with outgrowth of specific members of the airway microbiota.

## The IL-23/Th17 Axis in Chronic Inflammatory Disease

IL-17-producing CD4 T helper (Th17) cells were recognised as a discrete population of CD4^+^ T cells following the identification of their role in the pathogenesis of experimental autoimmune encephalomyelitis (EAE), a preclinical model of multiple sclerosis (MS) [26, 27]. Subsequently, these cells were found to be key orchestrators of numerous autoimmune and chronic inflammatory diseases [28]. In addition, several innate and adaptive immune cell populations were identified as sources of various IL-17 family members that play important roles in diverse inflammatory settings (Table 1).

**Table 1:** Cellular sources of IL-17 family members identified in distinct chronic inflammatory diseases. Abbreviations: ILC3, type-3 innate lymphoid cells; NKT, natural killer T cells

**Cellular sources of IL-17 cytokines**			
**Disease setting**	**Cytokine**	**Cell source**	**Reference**
Asthma	IL-17A IL-17E/IL-25	CD4 T cells Neutrophils Eosinophils	[29, 30]
Idiopathic pulmonary fibrosis	IL-17A, IL-17B	CD4 T cells γδ T cells Alveolar macrophages	[31, 32]
Chronic obstructive pulmonary disease	IL-17A	CD4 T cells Neutrophils	[33–38]
Cystic fibrosis	IL-17A, IL-17F	CD4 T cells γδ T cells NKT cells ILC3 Neutrophils	[29, 39, 40]
Bronchiectasis	IL-17A	CD4 T cells	[41]
Rheumatoid arthritis	IL-17A, IL-17B, IL-17E/IL-25	CD4 T cell γδ T cells	[42–51]
Multiple sclerosis	IL-17A, IL-17F	CD4 T cells CD8 T cells γδ T cells ILC3 Microglia Astrocytes	[52–58]

Th17 cells develop from naïve precursors following recognition of their cognate antigen and are polarised along the Th17 lineage in response to signalling by the innate cytokines IL-1, IL-6 and TGFβ [56]. These cells are characterised by expression of the “master” transcription factor retinoic acid receptor-related orphan receptor gamma (RORγt) which, in turn, promotes expression of a number of key Th17 cell-associated receptors, including IL-23R and CCR6. Th17 cells produce IL-17A and IL-17F along with a range of other cytokines including IL-21, IL-22, TNFα, and IL-10. IL-17A and IL-17F signal through a heterodimeric receptor complex composed of the ubiquitously expressed IL-17RA and the non-hematopoietic cell-restricted IL-17RC that, in the airways, is expressed on epithelial cells and fibroblasts [59]. IL-17-receptor engagement induces epithelial cell expression of anti-microbial peptides and production of CXC chemokines and granulopoeitic factors that are critical in the recruitment and activation of neutrophils which, collectively, contribute to protection against infection by extracellular bacterial and fungal pathogens [60, 61]. Th17 cells also contribute to the maintenance of barrier function at mucosal sites [62]. It is clear, therefore, that microbial stimulation and Th17 cell function are closely associated and, in fact, Th17 cell development is diminished in germ-free animals, with a concomitant decrease in susceptibility to many preclinical models of complex diseases such as MS, uveitis, RA and psoriasis [63–66].

Self-antigen-specific Th17 cells that drive many autoimmune and chronic inflammatory diseases develop in the same manner as those elicited upon infection, but appear to be innocuous immediately following differentiation [54, 56, 67, 68]. In order for self-reactive Th17 cells to gain pathogenic potential—the ability to migrate to and access tissue sites where self- or autogenous antigens are expressed and, therein, orchestrate the inflammatory response—these cells require exposure to the cytokine IL-23, which regulates their conversion to pathogenic effectors of chronic inflammation. Upregulation of IFNγ, GM-CSF and other inflammatory mediators in IL-23-stimulated Th17 cells appears to be associated with pathogenicity and suggests the role of Th17 cells extends beyond the actions of IL-17. Hence, these so-called ex-Th17 cells (lately referred to as Th17.1 cells in human studies) are activated and develop differently to those that protect against acute infection with, for example, *Candida albicans* or *Salmonella enterica* that do not require IL-23 signalling or deviation of Th17 cell cytokine expression to mediate their effector functions [54, 69, 70]. Whether or not IL-23 signalling is critical for the effector function of Th17 cells that contribute to chronic inflammatory diseases of the airways such as pulmonary fibrosis, CF, COPD and asthma is not known. In fact, very little is known about the influence of the microbiota that colonises the respiratory tract on the expression of IL-23 and other molecules that drive Th17 cell-mediated immunity locally or systemically.

## The Centricity of IL-17-Mediated Immunity in the Airways

It is clear that the microbiome is an important determinant of human health and that the host response to its microbial symbionts at several mucosal sites has a significant impact on chronic inflammation. Our understanding of host-microbe interactions in the airways is very much at an early stage, but increasing reports link this microbiota with a range of pulmonary and extra-pulmonary disorders. Central to many of these findings is the role of IL-17-producing immune cells and their activation by innate cytokines, including IL-1, IL-6 and IL-23. In the following sections, we summarise what is known about the induction of IL-17-driven immunity by respiratory tract symbionts, and their potential role in the development and resolution of inflammation in the airways, and beyond.

## Th17-Type Immunity in Chronic Respiratory Diseases

### Asthma

Asthma is a chronic inflammatory disease of the airways associated with airway hyperresponsiveness (AHR), leading to bronchoconstriction and airflow obstruction. In asthma patients, exposure to inhaled allergens leads to excessive activation of an inflammatory response in the lungs, typically mediated by allergen-specific Th2 cells that produce IL-4, IL-5 and IL-13, and which coordinate an allergic response centred around eosinophil recruitment and allergen-specific IgE production. Several murine studies have demonstrated a crucial role for commensal microbes in maintaining respiratory homeostasis. In many different models of allergic (or eosinophilic) asthma, AHR, local Th2 cytokine production, IgE concentrations and airway eosinophilia are all elevated in mice housed in germ-free conditions or administered antibiotics in early life, compared to conventionally housed mice or untreated controls [71–75]. Recolonisation of germ-free mice with a normal microbiota appears to suppress systemic IgE production by B cells and downstream expansion of basophilic precursor cells in the bone marrow; otherwise primed to promote a hypersensitive allergic response upon allergen exposure [74]. Numerous studies have demonstrated how stabilisation of the developing microbiota in neonatal mice induces CD4^+^CD25^+^Foxp3^+^ regulatory T (Treg) cells that decrease responsiveness toward aeroallergens; an effect that persists into adulthood [71, 75, 76]. Respiratory symbionts, specifically, have been reported to provide protection against allergic asthma. Adult mice whose lungs were exposed to *S. pneumoniae* before or after sensitisation with the model allergen ovalbumin (OVA) developed a regulatory-type immune response, characterised by elevated numbers of FoxP3^+^ Treg cells in lung-draining mediastinal lymph nodes and allergen-specific IL-10 secretion, associated with significantly reduced Th2 cytokine production, eosinophil recruitment and serum IgE [77]. Of note, only exposure to *S. pneumoniae* at OVA challenge led to a reduction in AHR, perhaps indicating that the mechanisms that suppress this response are transient and depend on recent bacterial stimulation.

Notwithstanding these findings, the adult human asthmatic airway has been associated with increased burden of *Haemophilus, Moraxella, Neisseria, Staphylococcus* and *Streptococcus* taxa, and a reduction in *Veillonella* and *Prevotella* species [5, 78–83]. URT colonisation by *S. pneumoniae, H. influenza* or *M. catarrhalis* within the first month of life was associated with increased risk of childhood asthma, and susceptibility to exacerbations in both mild and severe forms of disease [84, 85]. Circulating eosinophil counts and total IgE were significantly increased in children colonised neonatally with any or a combination of these bacteria, by the time they reached 4 years of age [84]. Interestingly, URT colonisation of these neonates with *H. influenzae* or *M. catarrhalis* engendered a mixed Th1/Th2/Th17 response, while *Staphylococcus aureus* colonisation induced a Th17-specific inflammatory profile [86].

Approximately 10% of asthmatics develop a neutrophilic form of disease driven by Th17-type inflammation, which is generally associated with a more severe disease phenotype and resistance to steroids [87, 88]. In these patients, heightened IL-17 expression and neutrophil infiltration can be detected in lung tissue, induced sputum, and serum, and it appears that neutrophils and eosinophils may also be an important source of IL-17 that sustains airway inflammation [29, 30, 87–89]. Neutrophilic asthma appears to be associated with a microbial profile quite distinct from that of eosinophilic asthma, with overall reduced microbial diversity and increased presence of opportunistic pathogens [90]. Increased burden of Proteobacteria species, including *H. influenza* and *M. catarrhalis,* routinely correlate with neutrophilic asthma, resulting in heightened Th17-related gene expression, CXCL8 production and airway neutrophilia [78, 82, 91, 92]. Patients with poorly controlled neutrophilic asthma exhibited high abundance of *Haemophilus* species and generally low bacterial diversity [93].

Murine studies have helped delineate how these diverse disease phenotypes may result from the differential induction of Th17-type immune responses. For example, it was found that, unlike the Treg-mediated protective responses elicited by *S. pneumoniae* in adult mice, neonatal exposure to *S. pneumoniae* or *H. influenzae* can trigger exacerbated allergic asthma in adulthood, associated with increased Th17 and Th2 cytokine secretion, and enhanced infiltration of neutrophils and eosinophils into the lungs [94, 95]. Intriguingly, Yang and colleagues used a model of long-term colonisation of the airways by *H. influenzae* to demonstrate the conversion of a steroid-sensitive Th2 model of eosinophilic inflammation to a steroid-resistant Th17-driven form of neutrophilic disease [96]. A significant increase in expression of IL-17 and RORγt was detected in the lungs of mice exposed to *H. influenzae*, which was also associated with increased mucin production and airway remodelling. Separately, it was reported that infection with *H. influenzae* during sensitisation alone can promote this conversion from eosinophilic to neutrophilic inflammation in the airways, underscoring the enduring impact of stimulation by *H. influenzae* on the development of airway inflammation [97]. Respiratory infection with *M. catarrhalis* similarly exacerbated a house dust mite (HDM) model of allergic asthma in an IL-17- and TNFα-dependent manner [98]. Airway inflammation was alleviated, and goblet cell hyperplasia and mucus production reduced in IL-17-deficient allergic mice infected with *M. catarrhalis*, compared to uninfected controls, confirming the crucial role of Th17-type inflammation in this response.

**Fig. 1:**
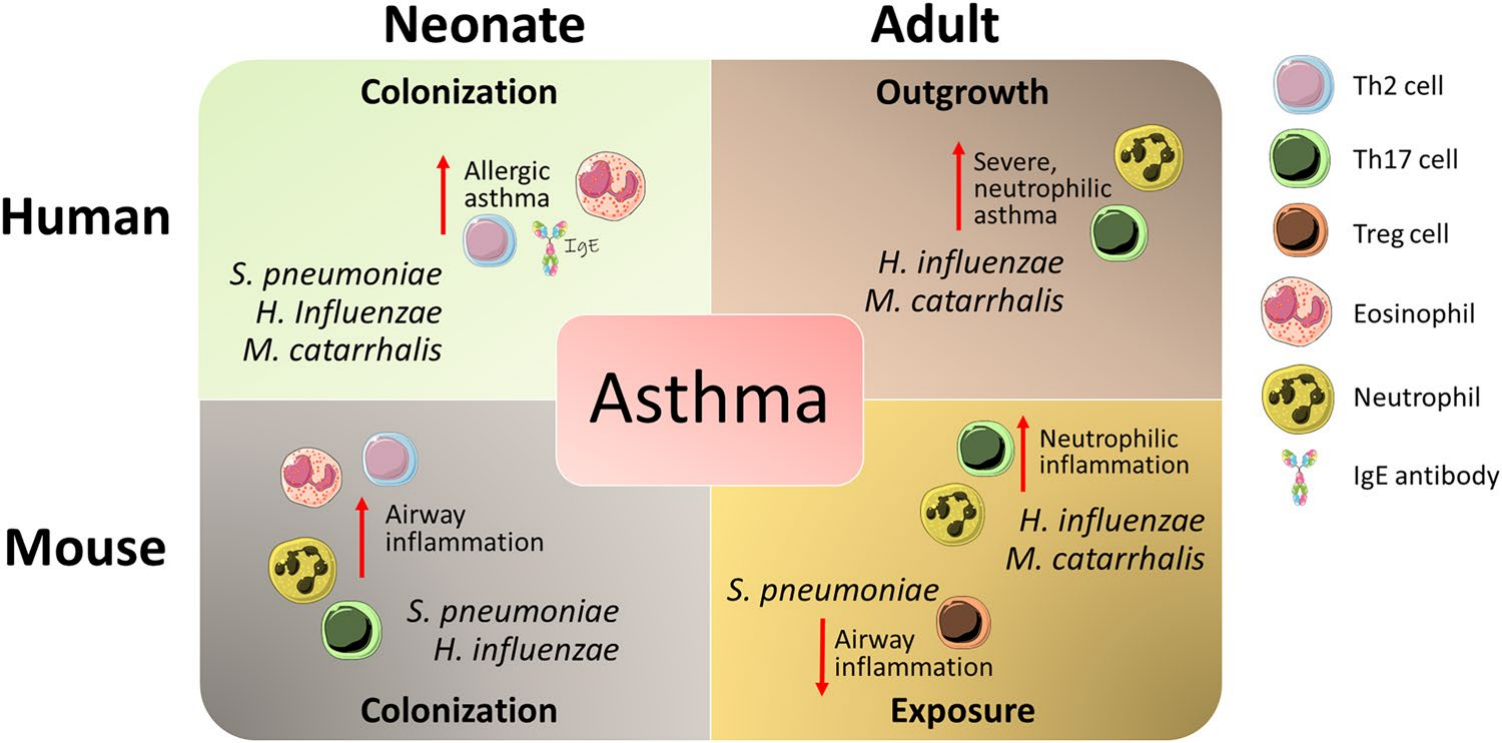
Inflammatory and suppressive T cell populations and granulocyte involvement in allergic airway inflammation has been associated with acute or long-term exposure to discrete bacterial species. Reduced microbial diversity in the lungs and outgrowth of specific taxa is associated with IL-17-type inflammation in the airways, and systemically. Relative changes in bacterial abundance and their association with cellular mediators of inflammation are indicated

Together, these studies indicate that allergen-specific T cells can be skewed into distinct suppressive or inflammatory phenotypes depending on the bacterial species encountered and the temporality of that exposure (Fig. 1); with individual lymphocyte subpopulations having a significant influence in determining clinical manifestation and disease severity in asthma. Notably, pre-clinical models that rely on peripheral sensitisation may artificially promote a type-2 immune response, whereas allergic-sensitisation through the airways, as occurs in humans, more likely involves Th17-dominant immunity [99].

Finally, a role for IL-23 and Th17-type immunity in allergic asthma has recently been described. Upon IL-23 airway exposure, Th2 cytokine production, eosinophil and neutrophil recruitment to the airways, goblet cell hyperplasia and AHR were all enhanced following allergen challenge [100, 101]. However, IL-17A only appeared critical for allergen-induced neutrophil recruitment into the airways. Separately, it was reported that exogenous IL-23 administration to the airways, in the absence of any allergen, can lead to eosinophilia and AHR, mimicking the non-allergic eosinophilic asthma (NAEA) phenotype [102]. This may be relevant given that pathogenic Proteobacteria often enriched in the airways of patients with asthma or COPD, including *Haemophilus influenzae* and *Moraxella catarrhalis,* induce significantly more IL-23 by host innate immune cells, compared to *Prevotella* species commonly found in healthy lungs [103].

## Chronic Obstructive Pulmonary Disease

Chronic obstructive pulmonary disease (COPD) is characterised by chronic bronchitis, mucociliary dysfunction, emphysema and a progressive decline in lung function. Exposure to tobacco smoke is the main environmental risk factor for the development of COPD, but acute exacerbations commonly result from bacterial and viral infections [104]. While evidence that smoking results in significant changes to the lung microbiome is limited, it does lead to changes in that of the URT [6, 17]. This may play an important etiological role in COPD because it has been demonstrated that microaspiration of disordered URT bacteria to the otherwise healthy lung can lead to an imbalance in the lung microbiome [105]. This, in turn, is associated with increased subclinical lung inflammation characterised by elevated Th17 responses, neutrophil accumulation and altered TLR4 signalling in alveolar macrophages, together with an increase in exhaled nitric oxide [105, 106].

Indeed, changes in the lung microbiota have been associated with the immunopathogenesis of COPD. Notwithstanding inter- and intra-subject heterogeneity, COPD patients, even when clinically stable, have a microbiome that lacks the diversity and richness of that of a healthy individual [5, 107]. A reduction in *Prevotella* and *Veillonella* and an overrepresentation of *Actinobacteria* and *Proteobacteria* species, in particular *Haemophilus, Moraxella* and *Pseudomonas,* have been reported in COPD patients [6, 108, 109]. These shifts in the composition of the microbiome appear to be strongly associated COPD exacerbation [107, 110, 111].

Th17 cells have been implicated as important inflammatory mediators in COPD with increased IL-17-expressing CD4 T cells and concentrations of related cytokines detected in the bronchial submucosa, airway epithelium, lung tissue, BALF and peripheral blood of COPD patients, compared to smokers without COPD and their healthy counterparts [33–37]. Neutrophilic infiltration mediated by IL-1β and IL-17 is prominent in COPD and increased recruitment correlates with a worsening disease course [112, 113]. Interestingly, the degree of neutrophilic inflammation was recently linked to Haemophilus-driven immune responses in the lungs [110]. Genetic predisposition may underscore these responses too, since polymorphisms in the *Il17a* gene have been associated with the risk of COPD related to tobacco smoking [114]. Notably, in a manner similar to that observed in other inflammatory conditions, conversion of Th17 cells to a more pathogenic IL-17^+^IFNγ^+^ double positive ex-Th17-like phenotype has been reported in COPD patients, with a correlation between increasing IFNγ^+^ CD4 T cell frequency and decreased lung function [38].

Using a murine model of chronic lung inflammation, which mimics many key features of COPD, microbiome dysbiosis involving a decrease in *Prevotella* and an increase in *Pseudomonas, Lactobacillus* and *Chryseobacterium* was observed in mice treated with LPS and elastase [115, 116]. Elevated levels of IL-1β and IL-6 were found in the BALF of diseased mice, coincident with the presence of IL-17A-expressing collagen- and elastin-specific CD4^+^ and γδ T cells in the lungs. The frequencies of these pulmonary IL-17A^+^ CD4 and γδ T cells was reduced in germ-free mice and in mice with an antibiotic-depleted microbiota [116]. Crucially, airway inflammation was ameliorated, and lung function improved in these mice, as well as SPF mice treated with an IL-17A-neutralising antibody. Separately, emphysema was attenuated in IL-17A- and IL-17RA-deficient mice exposed to long-term cigarette smoke, compared to wild-type controls, and IL-1RI–dependent IL-17A was found to be critical for pulmonary neutrophilia in *H. influenzae*-induced acute exacerbation using the same model [117–119]. While still preliminary, these studies support the idea that both chronic lung inflammation in COPD and the acute immunopathology linked to exacerbation are modulated by IL-17-centric host immune responses to a dynamic microbial environment.

## Cystic Fibrosis

Cystic fibrosis (CF) is an autosomal recessive disorder caused by a mutation in the cystic fibrosis transmembrane conductance regulator (CFTR) gene which results in thickened mucus secretions in the airways of the lungs and the ducts of the pancreas, and impaired mucocillary clearance [120]. Chronic pulmonary disease, associated with excessive neutrophilic inflammation and chronic microbial infection, is the most significant contributor to morbidity and mortality in CF [121]. The secretion of elastase and MMPs, including MMP9, by activated neutrophils contribute to a decline in pulmonary function [122, 123]. Expression of IL-23 and IL-17, which are crucial for neutrophil recruitment, is significantly elevated under steady-state conditions in the airways of CF patients, compared to controls [124–126]. Moreover, innate and memory T cell-derived IL-17, as well as neutrophil-derived IL-17, are significantly increased in CF airways during pulmonary exacerbation which, in turn, is associated with increased production of the pro-neutrophilic cytokines CXCL8, IL-6, G-CSF and GM-CSF [39, 125–128]. Importantly, Th17 cells have been identified in the airway submucosa of children with CF, early in the course of the disease, implicating Th17-driven inflammation in disease etiology [39].

Lung microbial dysbiosis plays a critical role in the pathophysiology of CF. Commencing in the first months and years of life, abnormally viscous secretions and dysregulated inflammation caused by the CFTR mutation are linked with an excessive bacterial biomass, but reduced diversity, in the lower airways [129, 130]. Total bacterial biomass is linked to features of early structural disease such as airway wall thickening, mucus plugging, bronchiectasis and parenchymal disease scores [131]. Conversely, reduced bacterial diversity and increased dominance of certain species, particularly *S. aureus* and *H. influenzae*, correlates with antibiotic use, age and heightened inflammation [7, 129, 132, 133]. From late childhood onwards *Mycobacterium abscessus*, *Burkholderia cenocepacia* and *Pseudomonas aeruginosa* begin to dominate and are strongly associated with disease progression [134–139]*.* Nevertheless, recent culture-independent studies have found that both the early stage and adult CF lung is more polymicrobial than previously thought [140, 141]. While interpatient heterogeneity exists, *Rothia, Gemella, Actinomyces, Stenotrophomonas, Neisseria* and *Streptococcus* species as well as the obligate anaerobes *Prevotella, Veillonella* and *Fusobacterium* have been commonly detected in CF airways [140–148]. The functional implications of these diverse and variable non-classical CF bacteria require further study.

Elevated IL-23 and IL-17—in steady-state conditions and during exacerbations—are found in the sputum of CF patients colonised with *P. aeruginosa,* but not *S. aureus*, and decrease upon antibiotic use [124, 126, 149, 150]. Neutrophils in the sputum and blood of CF patients experiencing *P. aeruginosa* exacerbation express IL-17A and the inducible IL-17RC receptor subunit, in an IL-23-dependent fashion, and are associated with elevated elastase and MMP9 activity, compared to post-antibiotic treatment neutrophils [150]. In mice challenged with *P. aeruginosa*, deficiency in the *Cftr* gene results in elevated IL-17 concentrations in BALF, associated with enhanced expression of IL-6, IL-8 and CXCL2 and increased neutrophil counts, compared to *P. aeruginosa-*infected WT controls [151]. Neutralisation of IL-17A leads to reduced neutrophilia and lung pathology. Similarly, IL-23-deficient mice challenged with a clinical, mucoid isolate of *P. aeruginosa* developed significantly less airway inflammation than WT mice, associated with reduced IL-17, IL-6 and CXCL1 and decreased airway neutrophilia and MMP-9 expression [152]. Interestingly, bacterial clearance was not diminished in these mice, indicating that IL-23 may be a promising therapeutic target to reduce airway damage in patients with CF.

## Non-CF Bronchiectasis

Chronic pulmonary infection and sustained neutrophilic inflammation also feature strongly in non-CF bronchiectasis (henceforth bronchiectasis), which varies in etiology but is characterised by abnormal, thick-walled and dilated bronchi, reduced lung function and the production of purulent sputum [153, 154]. While the majority of cases of bronchiectasis are idiopathic, a significant number present as a smoking-related COPD co-pathology [155]. Bronchiectasis is less well defined than CF but studies of the microbiome suggests that a core airway microbiome is shared between pediatric CF and bronchiectasis patients, which diverges in adulthood [156]. *Haemophilus, Pseudomonas, Streptococcus, Staphylococcus, Moraxella, Veillonella, Prevotella* and *Achromobacter*, as well as non-tuberculous *Mycobacterium*, are amongst the most abundant bacterial taxa in the lungs of patients with bronchiectasis [154, 157–160]. Moreover, as with CF, significantly elevated IL-23 and IL-17 production is seen in children and adults with bronchiectasis, which is reduced following treatment with the antibiotic clarithromycin [39, 41, 161]. IL-1α, IL-6 and CXCL8 levels were also significantly elevated in adults with established bronchiectasis and concurrent airway infection [41].

## Idiopathic Pulmonary Fibrosis

Idiopathic pulmonary fibrosis (IPF) is a progressive and usually fatal fibrotic lung disease of unknown etiology that is associated with chronic inflammation and dysregulated wound-healing [162]. It appears that IL-1β and IL-23-driven IL-17 signalling is instrumental early in the pathogenesis of pulmonary inflammation and fibrosis, likely through promotion of neutrophilic inflammation and MMP expression by fibroblasts [32, 163–165]. Indeed, elevated neutrophil counts in BAL fluid is a predictor of early mortality in IPF patients [166].

Recent studies have supported a role for the lung microbiota in IPF pathogenesis, and germ-free mice are protected against mortality in preclinical models of disease [167, 168]. In addition, a number of anti-microbials have entered clinical trials in human patients with IPF [169, 170]. Characterisation of bronchoalveolar lavage fluid (BALF) from IPF patients has demonstrated that disruption of the airway microbiota is associated with alveolar inflammation and significantly increased risk of disease progression and mortality [167, 168, 171]. Specifically, increased abundance of *Neisseria, Haemophilus*, *Veillonella, Staphylococcus* and *Streptococcus* taxa have been associated with worsening IPF [171–173].

The precise mechanisms by which the lung microbiota influences IPF pathogenesis, and the role of IL-17 is only beginning to be revealed. Of note, it was recently demonstrated that microbiota-induced IL-17R signalling directly induces production of proinflammatory and profibrotic genes in lung epithelial cells and promotes lung fibrosis in a bleomycin-induced mouse model of IPF [31]. In that study, IL-17B was produced by alveolar macrophages correlating with the significantly elevated IL-17A and IL-17B expression by human BALF cells from IPF patients, compared to controls [31, 32]. Germ-free or antibiotic-treated mice, as well as mice that are deficient in IL-17A, IL-17B or IL-17E/IL-25, are resistant to bleomycin-induced fibrosis [31, 32, 163]. Importantly, Yang and colleagues identified outer membrane vesicles (OMVs) expressed by respiratory tract-associated Gram-negative anaerobes, including *Bacteroides ovatus, Bacteroides stercoris* and *P. melaninogenica*, that induce IL-17A and IL-17B expression, Th17 cell development and neutrophil recruitment to the lungs [31]. Evidence indicates that expansion of these specific species may result from altered host metabolic pathways following lung injury. Together, these findings support the idea that dysregulated growth of specific respiratory symbionts could initiate or perpetuate the immunopathology of IPF and opens a new area of investigation in the etiology of this poorly understood disease. If confirmed by independent investigators, the mechanistic discoveries by Yang and colleagues may point to novel microbial and metabolic targets for early therapeutic intervention in IPF.

## Systemic Impact of the Airway Microbiota

### Oral and Lung Bacteria Promote Joint Inflammation

Rheumatoid arthritis (RA) is a systemic autoimmune disease that is particularly associated with chronic inflammation at synovial joints. Pathogenic Th17 cells orchestrate the destruction of articular cartilage and underlying bone via expression of the pro-osteoclastogenic cytokines IL-1, IL-6, IL-17 and TNFα and B cells produce autoantibodies against a variety of endogenous antigens [42–44, 174–176]. IL-23 is critically required for pathogenicity of collagen-specific Th17 cells in the pre-clinical model collagen-induced arthritis (CIA) and pathogenic human Th17.1 cells (CD161^+^ Th17 cells that have converted to an IFNγ-producing phenotype) are important in the early phase of RA [177–179]. IL-23 also promotes expression of IL-1, IL-6, IL-17, GM-CSF and TNFα in synovial tissues and has been shown to stimulate osteoclast differentiation in human PBMCs in an IL-17-dependent manner [177, 178, 180].

**Fig. 2:**
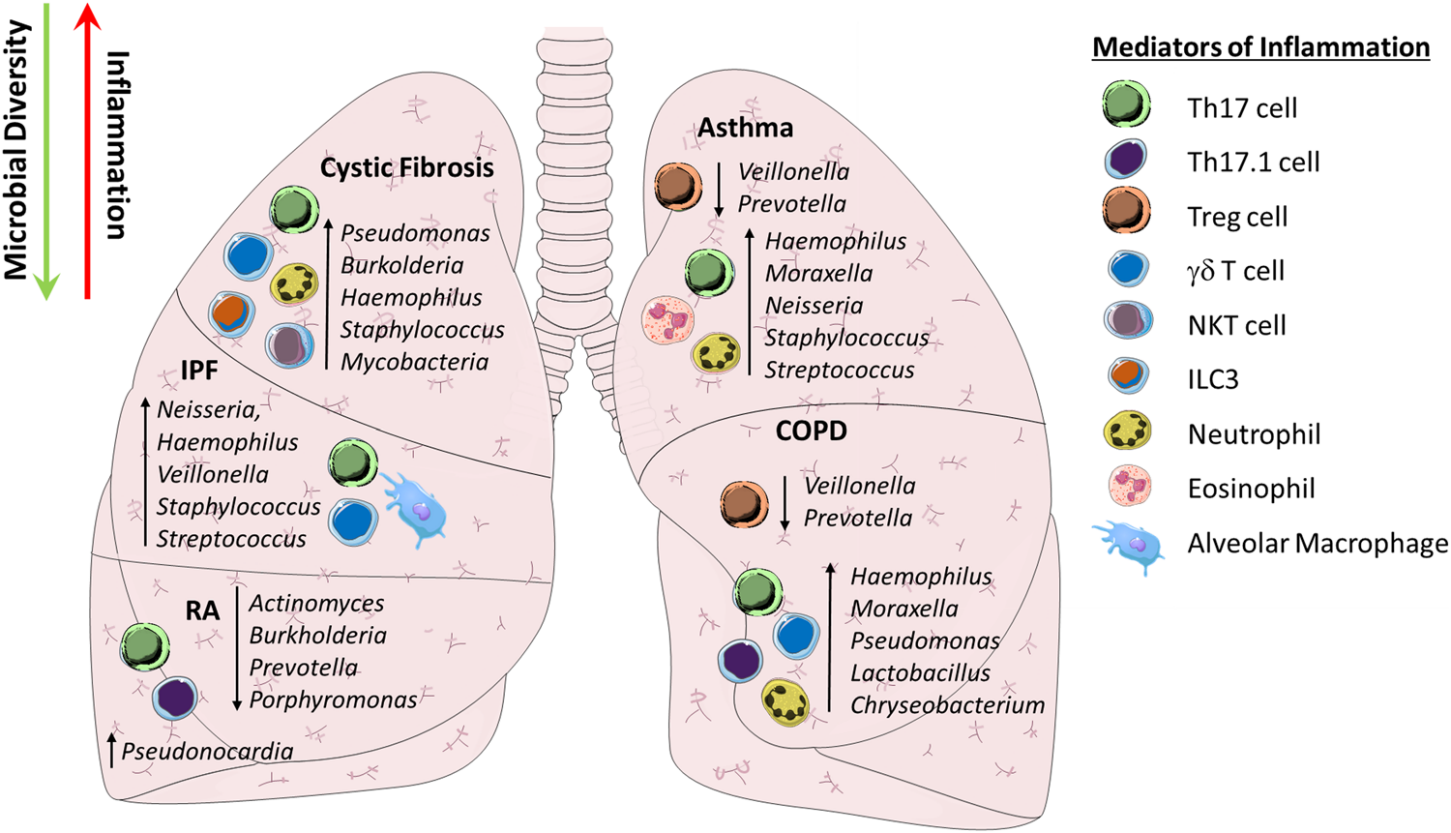
Relative changes in bacterial taxa and inflammatory mediators during chronic inflammatory disease. Reduced microbial diversity in the lungs and outgrowth of specific taxa is associated with IL-17-type inflammation in the airways, and systemically. Relative changes in bacterial abundance and their association with cellular mediators of inflammation are indicated. Abbreviations: COPD, chronic obstructive pulmonary disease; IPF, idiopathic pulmonary fibrosis; ILC3, type-3 innate lymphoid cell; NKT, natural killer T cell; RA, rheumatoid arthritis

Th17 cells also promote humoral immunity through germinal center formation, B cell activation and isotype class switching [181]. RA is characterised by increased levels of circulating autoantibodies including rheumatoid factor (RF) and anti-citrullinated protein antibodies (ACPA) [42]. Systemic RF and ACPA can be identified several years before the onset of synovial pathology, suggesting that immunity against these proteins is initiated outside the joint [182]. A concordance rate as low as 15% in monozygotic twins indicates a strong environmental influence in RA development and IgA-dominant autoantibody responses in individuals with preclinical and early RA support that the initiation of RA-related autoimmunity might occur at mucosal sites [65, 183–185]. Indeed, growing data indicates that the microbiome may be an important factor. Disease is attenuated in numerous pre-clinical models when mice are housed in germ-free conditions [186–188]. Antimicrobial drugs have proven an effective treatment in some RA patients for decades [189]. And, it has recently been reported that active disease correlates with increased intestinal burden of *Prevotella copri*, *Lactobacillus salivarius* and the Actinobacteria genius *Collinsella* [190–193]. Similarly, in the lower airways, microbiome analysis of BAL samples from preclinical or early, untreated RA patients found significantly reduced microbial diversity compared to healthy controls, with a decrease in the relative abundance of the genera *Actinomyces*, *Burkholderia*, *Prevotella* and *Porphyromonas* [8, 194] (Fig. 2).

Autoantibodies have been detected in sputum from preclinical high-risk individuals—even in the absence of seropositivity [185]. Elevated ACPA prior to RA diagnosis has also been linked to bronchiectasis and asthma, while cigarette smoke and other bronchial stressors—perhaps including microbial factors—can increase ACPA titers and susceptibility to RA in persons with disease-associated HLA alleles [185, 195–198]. Significantly more ACPA-positive early RA patients had germinal center formation in bronchial tissue and histopathologic evidence of airway inflammation, compared to ACPA-negative RA patients or healthy controls, regardless of smoking status [199]. And, inducible bronchus-associated lymphoid tissue (iBALT)—not present in healthy human lung tissue—containing RA autoantibody-producing plasma cells, was increased in lung biopsies from RA patients with related lung disease [200]. These studies strongly implicate the lungs as a critical mucosal site in the etiology of autoimmune arthritis.

Periodontitis and RA share similar pathophysiological mechanisms including chronic inflammation with adjacent bone resorption. Not surprisingly then, a link has also emerged between oral microbiome perturbations, oral inflammation and arthritogenesis. *Rothia, Lactobacillus* and *Prevotella* species are enriched in the oral microbiome of pre-articular high-risk individuals, while *Haemophilus* and *Neisseria* species were depleted [192, 201]. Oral pathobionts including *P. nigrescens* or *Porphyromonas gingivalis* have also been associated with increased ACPA titers in patients with RA. Importantly, *P. gingivalis* was demonstrated to preferentially drive the generation of Th17-type immunity in human peripheral blood mononuclear cells (PBMC) by stimulating production of IL-1β, IL-6 and IL-23, but not the Th1-associated cytokine IL-12 [202]. And, in mice, oral administration of either *P. nigrescens* or *P. gingivalis* exacerbated CIA severity via IL-1-driven collagen-specific Th17 responses [203–205]. Finally, DNA fragments from *P. nigrescens, P. gingivalis* and *Prevotella intermedia,* and antibodies against periodontal bacteria, have been identified in serum and synovial fluid from RA patients with periodontitis [206, 207]. Collectively, these findings support the involvement of Th17-promoting URT pathobionts in the pathogenesis of RA. It may be that, in genetically susceptible individuals, bacteria residing in diverse regions of the respiratory tract can provide the environmental trigger leading to airway inflammation and, ultimately, systemic autoimmunity.

### Regulation of CNS Autoimmune Inflammation in the Airways

Multiple Sclerosis (MS) is a chronic, progressive inflammatory disease of the central nervous system (CNS). MS is instigated by the infiltration of autoreactive T cells and other immune cell subsets into the CNS which attack the myelin sheath surrounding nerve axons, resulting in the formation of an inflammatory plaque and reduced signal conductance [208]. The etiology of MS is not fully understood and much of our understanding of the pathophysiology of the disease has arisen from studies of experimental autoimmune encephalomyelitis (EAE), an animal model that recapitulates many of the clinical and pathological features of the human disease. These investigations have revealed that Th17 cells are the critical pathogenic effector cells in EAE and that exposure to IL-23 is a critical step in their acquisition of pathogenic Th1-like properties [209, 210]. In MS patients too, CD161^+^ Th17.1 cells that express IFNγ and GM-CSF have been demonstrated to be the main pathogenic T cell subset associated with relapse and disease progression [211].

Epidemiological studies have long associated relapses in MS patients with systemic infection. Correale and colleagues demonstrated that bystander activation and increased sensitisation of autoreactive myelin-specific T cells resulted in elevated numbers of circulating IFNγ^+^ CD4 T cells and exacerbated disease in relapsing–remitting MS (RRMS) patients shortly following systemic infection [212]. Originally identified as Th1 cells, it is possible that these cells are Th17.1 cells that have converted to a pathogenic phenotype following microbial stimulation. In mice, over 90% of IFNγ-producing CD4 T cells in the CNS at the height of disease are actually ex-Th17 cells that upregulated Tbet following exposure to IL-23 and now exhibit Th1-like properties [54]. Myelin-reactive Th17 cells only upregulate IL-23R following differentiation in peripheral lymphoid tissues, however, and are innocuous immediately following their differentiation in vitro and in vivo [56, 67]. When and where these cells are exposed to this pathogenic signal is unknown.

Studies in pre-clinical models have implicated the respiratory tract in the pathogenicity of CNS autoimmune inflammation. Using intra-vital microscopy in a rat model of disease, Odoardi and colleagues demonstrated that transferred myelin-reactive CD4 T cells migrate to the lungs and BALT prior to their appearance in the CNS and the onset of neurological deficits [213]. These cells appeared to reside transiently in bronchi and alveoli before accumulating in BALT and draining lymph nodes, during which time they under-went functional reprogramming that allowed them to enter the CNS and instigate neuroinflammation. Separately, it was found that expansion of a pro-inflammatory population of granulocytic myeloid-derived suppressor cells (MDSCs) in the lungs during EAE promotes Th17 cell pathogenicity in an IL-6-dependent fashion [214].

Conversely, the respiratory pathogen *Bordetella pertussis* was found to suppress the severity of disease in a mouse model of EAE [215]. Infection with *B. pertussis* resulted in increased numbers of IL-10-producing Treg cells that suppressed expression of the adhesion molecules VLA-4 and LFA-1 on myelin-specific Th17 cells, impairing their migration to the CNS. In addition, subclinical pulmonary inflammation induced by LPS exposure or in an autophagy-defective mouse model appears to stall the trafficking of CCR6^+^ Th17 cells in the lungs, through enhanced expression of the chemokine CCL20 [216].

Regardless of the clinical outcome, these studies identify the lungs as a critical site that impacts Th17 cell encephalitogenicity and disease exacerbation in MS. Germ-free or antibiotic-treated mice are relatively resistant to EAE and reconstitution of these mice with bacteria that specifically colonise the murine gut can promote Th17 cell pathogenicity; although a systematic review of relevant human studies failed to find major differences in the gut microbiomes of MS patients and healthy controls [217, 218]. Studies on an etiological connection between human respiratory tract bacteria and CNS autoimmune inflammation have not been reported but should now be a vital new avenue of MS research.

## Concluding Remarks

An axis exists between the airway microbiome and IL-17-type host responses that contribute to the immunopathology of many chronic inflammatory diseases. Advanced sequencing technologies have expanded our awareness of the respiratory tract microbiota which, during health, is low in biomass but high in diversity, and is dominated by Prevotella and Veillonella species [12, 13, 15, 17]. Reduced bacterial diversity is a common feature during exacerbation of chronic lung diseases such as asthma, CF, IPF and COPD, and often correlates with increased burden of discrete Proteobacteria species. Furthermore, whether by molecular mimicry, shared antigenic targets or epitope spreading, or through bystander activation and migration of self-reactive T cells, it appears that local inflammation at distinct sites along the respiratory tract can modulate extra-pulmonary diseases including RA and perhaps MS. Studies of the gastrointestinal tract demonstrate the strong influence that various mucosal symbionts exert over Th17 cell development and effector function, locally and systemically. Yet, we have scant understanding of the role played by individual respiratory tract bacteria, or those that contribute to a collective outcome, in shaping Th17-mediated chronic inflammation. It is a fascinating thought that our poor grasp of the early pathogenesis of multi-factorial diseases such as asthma, IPF and RA may lie in insufficient appreciation of the impact of the bacteria that colonise the respiratory tract and that, perhaps, these processes will become better elucidated with more detailed knowledge of how discrete respiratory symbionts promote pathogenic Th17-type immune responses locally in the airways, and systemically.

**Fig. 3:**
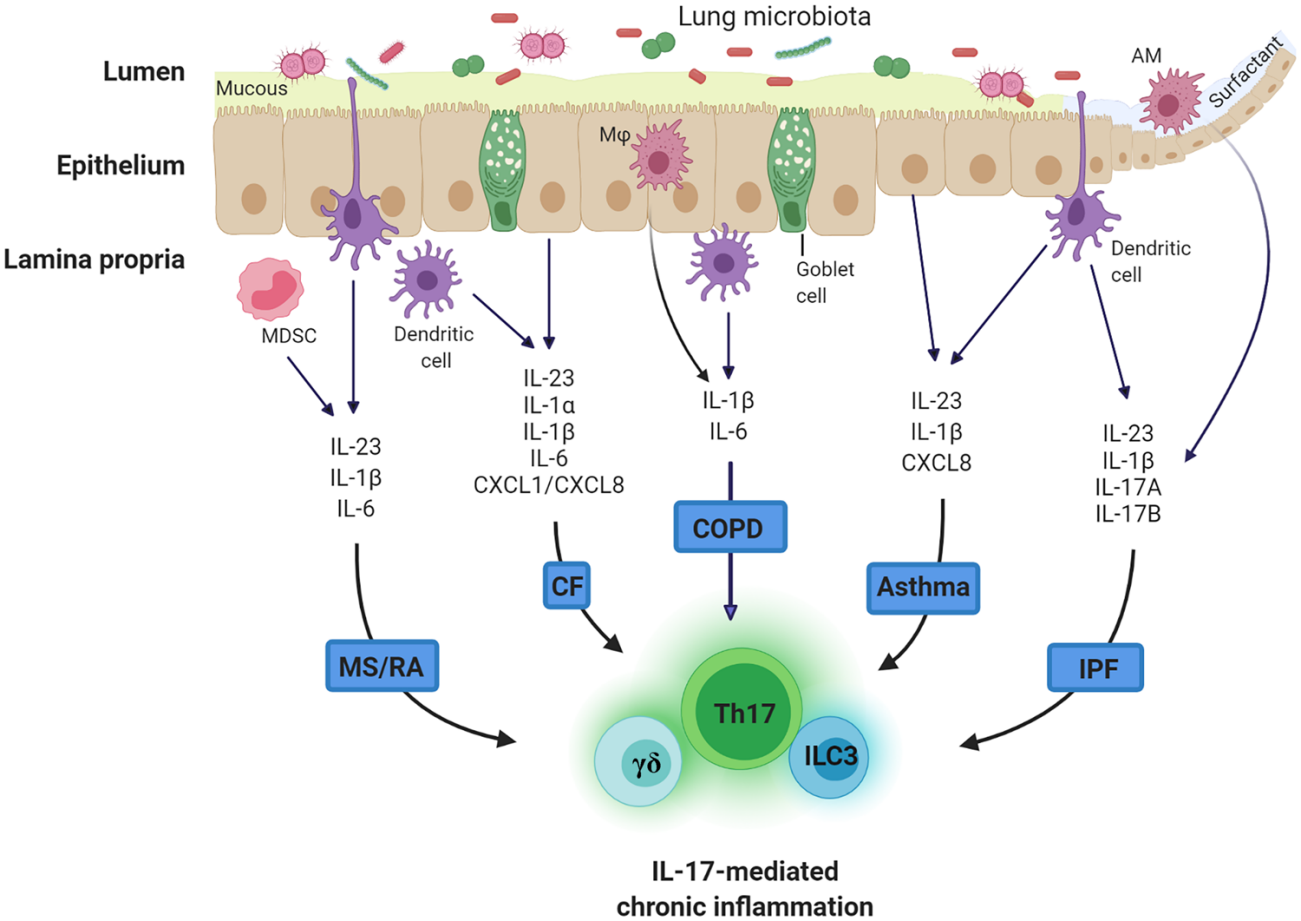
The centricity of IL-17-mediated immunity in chronic inflammation associated with the airways. Abbreviations: MDSC, myeloid-derived suppressor cells; AM, alveolar macrophage; $\gamma\delta$, gamma delta T cell; ILC3, type-3 innate lymphoid cell; CF, cystic fibrosis; COPD, chronic obstructive pulmonary disease; IPF, idiopathic pulmonary fibrosis; MS, multiple sclerosis; RA, rheumatoid arthritis RA. Created with BioRender.com

As our understanding of the respiratory tract microbiota progresses from description of this community to defining it’s impact on human health, it will be crucial to determine a causal role for specific bacterial strains in driving IL-17-signalling, neutrophil infiltration and resultant immunopathology (Fig. 3). Conversely, the metabolic changes associated with inflammation may create an environmental niche that some Gammaproteobacteria have developed the capacity to exploit, perpetuating a cycle of inflammation [219]. Hence, production of reactive oxygen species (ROS) and reactive nitrogen species (RNS) during an inflammatory response may allow adaptable strains to bloom and outcompete resident bacteria that lack this capacity but, alas, these bacteria may not be the primary instigators of inflammation. Either way, it is imperative we understand the earliest inflammatory events in order to prevent the initial development of these chronic inflammatory diseases.

If certain strains of bacteria that have a fundamental involvement in the aetiology of a disease can be identified, then early microbiome sequencing of susceptible individuals and therapeutic targeting of the IL-17 pathway may present a novel preventative approach. Several exciting new drugs targeting the IL-17 axis have proven efficacious in the treatment of inflammatory disorders such as psoriasis and inflammatory arthritis [28]. However, Th17 cells act in pleiotropic ways to orchestrate immunity at diverse sites of inflammation. IL-1β and IL-23 stimulate expression of numerous inflammatory molecules, in addition to guiding Th17 cell development and effector function. There is redundancy amongst members of the IL-17 family; for example, IL-17A and IL-17F use the same receptor complex to stimulate expression of chemokines and various myelopoietic and granulopoeitic factors. And, Th17 cells support B cell activation, isotype class switching and high-affinity antibody production. Hence, the effects of Th17 cells extend beyond the actions of IL-17A and may explain why IL-17-blockade during exacerbations in some chronic inflammatory diseases often fail or deliver only partial responses. It may be, too, that we miss the acute IL-17-driven pathophysiology in some of these diseases which has given way to a cycle of inflammation and dysbiosis by the time symptoms present clinically.

Identification of individual strains of bacteria that drive the initial inflammatory events, or those that collectively engender chronic inflammation, may permit prophylactic targeting at the very earliest stages of disease development. Airway microbiome composition can act as a biomarker of potential early disease activity and allow for stratification of at-risk individuals. Altered microbiota structure and increased burden of causal strains may signal for interventional approaches including the use of novel small molecule drugs, aerosol delivery of probiotics or narrow-spectrum antibiotics, the use of vaccines or administration of synthetic bacteriophages that reduce harmful populations of bacteria. Ongoing research in this area should also elucidate critical endogenous signalling pathways and new targets for drug therapies. Importantly, recent research has focused on non-canonical pathways involved in the acquisition of pathogenicity by Th17 cells. These include the RNA helicase DDX5 that controls aspects of RORγt-mediated Th17 cell development and CDL5, a scavenger receptor with pattern recognition receptor (PRR) activity that is expressed by lung epithelial cells, Th17 cells and macrophages in inflamed tissues and acts as a negative regulator of the transition from a non-pathogenic to a pathogenic Th17 phenotype [220–222]. In individuals at risk of developing chronic inflammatory disease, prophylactically targeting molecules that regulate progression to a pathogenic Th17 cell phenotype, while sparing protective Th17 capacity, should maintain a balanced immune response without increased susceptibility to infection and, perhaps, lead to improved patient outcomes compared to those achieved therapeutically.
